# Hospitalization and death after nursing home-to-nursing home transfer

**DOI:** 10.1093/geroni/igaf122.3790

**Published:** 2025-12-31

**Authors:** Ana Montoya, Pil Park, Slim Benloucif, Matthew Davis, Julie Bynum

**Affiliations:** University of Michigan, Ann Arbor, Michigan, United States; University of Michigan, Ann Arbor, Michigan, United States; University of Michigan, Ann Arbor, Michigan, United States; University of Michigan, Ann Arbor, Michigan, United States; University of Michigan, Ann Arbor, Michigan, United States

## Abstract

Although some nursing home (NH)-to-NH transfers have been associated with negative outcomes, it is unclear whether they are associated with a higher risk of hospitalization/death among long-term NH residents. Paired cohort analysis was used to evaluate whether long-term residents who experience a transfer are at greater risk for hospitalization or death compared to residents who remain in the same facility. Data: 20% national Minimum Data Set (MDS) assessments linked to Medicare claims. Long-term NH residents who underwent a NH-to-NH transfer in 2019 were matched 1:1 with residents who did not transfer—matching variables included residing in the same NH and month. The primary outcomes were acute care hospitalization and all-cause death within 90 days following the transfer. A total of 1,595 matched resident pairs were analyzed. Hospitalization rates (127.9 vs 126.6 per 1000 residents, p = 0.92) and all-cause mortality (9.4 vs 13.2 per 1000 residents, p = 0.32) did not differ between the transfer and non-transfer groups. After adjusting for demographics (age, sex, race/ethnicity, marital status), dual eligibility status, activities of daily living (ADL) scores, cognitive function scores (CFS) and comorbidity scores, transfer status was not associated with a higher risk of 90-day hospitalization (RR = 0.99; 95%CI [0.82-1.19]) or death (RR = 1.40; 95%CI [0.72-2.72]). Among long-term NH residents, transfer to another NH was not associated with a higher risk of hospitalization or mortality within 90 days post-transfer, after accounting for resident characteristics and comorbidities. These findings suggest that NH-to-NH transfer when necessary, may not inherently increase acute health risks for this population.

